# Enhancing Multidisciplinary Team (MDT) Skills in the Recognition, Escalation and Management of Clinical Deterioration on an Old Age Psychiatry Ward: A Quality Improvement Project

**DOI:** 10.1192/bjo.2026.11158

**Published:** 2026-06-30

**Authors:** Gayatri Dhar, Jaskiran Matharu, Nidhi Patel, Ivan Shanley

**Affiliations:** Essex Partnership University Trust, Rochford, United Kingdom

## Abstract

**Aims:**

Patients admitted to Older Adult Psychiatric Wards often have significant comorbidities putting them at increased risk of clinical deterioration. As MDT morale in managing these patients varied greatly, this project aimed to improve confidence and knowledge in recognising physical deterioration, escalating concerns and initiating Immediate Life Support (ILS).

**Methods:**

A pre-intervention questionnaire was distributed to MDT, including nurses, healthcare assistants, student nurses and allied health professionals. The questionnaire assessed knowledge and confidence in recognising clinical deterioration, escalation pathways, contacting emergency services, situation-background-assessment-recommendation (SBAR), and initiating ILS. The questionnaires used a mixture of close-ended questions, Likert scale, predefined option selection and open-ended short-answer questions, to explore perceived challenges. An interactive teaching session was delivered, focusing on acute clinical signs, NEWS chart, escalation pathways, practical ILS skills and SBAR. A confluent patient scenario was paired with questions to encourage participants’ engagement and learning. A post-teaching questionnaire with the same format as the pre-teaching questionnaire was completed to reassess confidence, awareness, and perceived impact on patient safety.

**Results:**

16 staff-members completed the pre-intervention questionnaire and 13 completing post-intervention. Pre-intervention, 19% of staff rated themselves as confident (Likert score 4–5) in recognising physical deterioration, 25% in escalation of concerns and 20% in initiating ILS. Anxiety of “getting it wrong” was reported by over 70% of respondents being cited as a barrier to escalation. 38% felt current escalation and management processes were clear.

Pre-intervention, 69% reported knowledge of local escalation policies, ILS processes and paramedic handover. Post-intervention this had grown to 100%.

There was substantial improvement in confidence across all domains post-intervention.On recognising physical deterioration, 92% of respondents rated themselves as confident or very confident. On escalation and calling emergency services and on initiating ILS, confidence increased to 92% and 83% respectively. Confidence in delivering a comprehensive handover improved from 25% pre-intervention to 92% post.

Post-intervention, 100% agreed or strongly agreed that the intervention would improve patient safety on the ward. Across every domain there was growth in mean confidence scores (mean increase of a factor of 1.46).

**Conclusion:**

Team knowledge and confidence in managing and escalating the acutely unwell patient was significantly improved following targeted ward-based teaching. Following staff feedback requesting visual prompts, a second PDSA cycle is planned to introduce informative posters around the ward to reinforce key learning points.

